# Social Stigma Related to COVID-19 Disease Described by Primary and Secondary School Teachers and Adolescents Living With HIV in Western Kenya

**DOI:** 10.3389/fpubh.2021.757267

**Published:** 2021-11-17

**Authors:** Ashley Chory, Winstone Nyandiko, Celestine Ashimosi, Josephine Aluoch, Roxanne Martin, Whitney Biegon, Dennis Munyoro, Edith Apondi, Rachel Vreeman

**Affiliations:** ^1^Department of Global Health and Health System Design, Arnhold Institute for Global Health, Icahn School of Medicine at Mount Sinai, New York, NY, United States; ^2^Department of Child Health and Paediatrics, School of Medicine, College of Health Sciences, Moi University, Eldoret, Kenya; ^3^Academic Model Providing Access to Healthcare (AMPATH), Eldoret, Kenya; ^4^Moi Teaching and Referral Hospital, Eldoret, Kenya

**Keywords:** COVID-19, stigma, teachers, HIV, Kenya

## Abstract

**Introduction:** Understanding community members' knowledge, attitudes, and beliefs about the novel SARS-CoV-2 virus and the prevalence of associated stigma are critical steps for increasing accurate public health knowledge, encouraging uptake of preventative or mitigating health behaviors, and ultimately bringing the COVID-19 pandemic under control.

**Methods:** We conducted a one-time, phone-based assessment to assess the presence of perceived COVID-19 community stigma reported by Kenyan primary and secondary school teachers, as well as adolescents living with HIV. Participants were previously enrolled in an ongoing, cluster-randomized trial to evaluate the impact of multi-media teacher training on teachers' negative attitudes and beliefs around HIV. The SAFI Stigma Questionnaire, a validated tool to assess HIV-related stigma in this setting, was adapted to ask questions regarding the stigma and discrimination experienced or perceived during the COVID-19 pandemic.

**Results:** We enrolled 330 participants in this study, including 311 primary and secondary teachers (56% female, average age 36 years) and 19 adolescents living with HIV (57.89% female, average age 16.37 years). None of the adolescent participants reported witnessing or experiencing discrimination related to COVID-19, nor did they report losing financial and/or social support. In contrast, the teacher participants reported prominent social stigma experiences of various levels and related to COVID-19. Teachers in the intervention group, who had completed the multi-media training on HIV-related stigma, were significantly less likely to think that the community viewed COVID-19 as a dirty or shameful disease, and less likely to feel it was important to keep their COVID-19 infection a secret, compared to the teacher control group.

**Conclusion:** These findings suggest that COVID-19-related stigma may be prevalent in western Kenya and that interventions to reduce community-level stigma for HIV may also have a protective impact on other stigmatized infectious diseases such as COVID-19.

## Introduction

Stigma is a critical consideration in understanding the experiences of vulnerable groups during the COVID-19 pandemic. Previous studies from other infectious disease outbreaks have demonstrated that health-related stigma is experienced by individuals who are directly and indirectly affected by the disease, as well as those viewed as most vulnerable to infection (1–4). Health-related stigma has been shown to be a deterrent to protective behaviors, and has been commonly documented in HIV, TB and Ebola outbreaks, among others (5–8).

Communities affected by Ebola-related stigma have suffered from harassment, rejection, isolation, physical violence, and diminished quality of life (9). Individuals in contact with Ebola patients have also been victimized, experiencing damage to their properties and thus prevented from returning to their homes (9, 10). Moreover, health campaigns utilizing “no touch” and quarantine policies during Ebola outbreaks can contribute to the “othering” of individuals associated with Ebola, further facilitating community stigmatizing beliefs (11). For children orphaned by Ebola, community fears and associated stigma have led to significant social isolation, loss of housing and severe mental health challenges (11, 12). Similar experiences have been documented by communities impacted by HIV. HIV-related stigma has been associated with a decrease in testing uptake (13), lower medication adherence (14), higher levels of mental health challenges (15, 16), substance use and sexual risk-taking behaviors and violence (15–18). Similarly, stigma has been associated with lower testing and treatment-seeking behaviors among those living with TB, as well as challenges with TB contract tracing and outbreak investigations (19–23).

As the COVID-19 pandemic evolved in 2020, reports of social stigma and discrimination against people of certain ethnic backgrounds and anyone perceived to have been in contact with the virus quickly began to emerge (24–27). COVID-19 stigma has been influenced by the novelty of the disease and the fears associated with the unknown, a lack of knowledge about how the virus spreads and gossip that leads to rumors and myths (27). In addition, politicization of the roles played by particular countries or ethnic groups, with blame being ascribed through derogatory terms used in describing SARS-CoV-2 may have further exacerbated the related COVID-19 stigma (27). Moreover, stigma can undermine social cohesion, leading to isolation of groups in which the infectious disease is more likely to spread (27), and therefore may create even more challenges in the control of the pandemic, as compliance with public health guidance may be significantly impacted. There is a crucial need during infectious disease outbreaks to evaluate how and where stigma is perpetuated, understand the experiences of affected groups, and develop strategies to reduce stigma.

The extent to which stigma related to one infectious disease or stigmatized condition might carry over to another infectious disease such as COVID-19 has not been well-studied. We could find minimal data about the clustering of stigma experiences, intersectional stigma, or subsequent carry over within the sub-Saharan African setting. Strategies to combat HIV-related stigma, including social activism, empathy-promoting programs and resilience and knowledge building, have been tailored for use in mitigating Ebola and other infectious disease outbreak-related stigma, suggesting transferability of stigma-prevention skills (9, 28–30). Despite this, the carryover effects of interventions targeting stigmatizing beliefs and behaviors related to one condition have also not been well-studied.

The first COVID-19 case in Kenya was confirmed on March 13, 2020, followed by a nation-wide mandatory quarantine for individuals entering the country on March 25, 2020 and universal testing policy on March 29, 2020 (31). National curfews were instituted in early April 2020, followed by a strict national lockdown preventing movement throughout the country. Kenya announced a phased reopening plan in June 2020, lifting nation-wide lockdowns and allowing for resumed international travel, with nighttime curfews to remain in place. Additionally, significant economic downturn is widely reported as COVID-19 prevention measures and behavioral responses restrict activity in Kenya and its partners (32, 33). Kenya has experienced several case surges, with an ~1,000 daily new confirmed cases that began at the end of November (34). As of January 28, 2021 there were 100,323 confirmed cases of COVID-19 throughout Kenya, with 71,254 recovered patients and 1,751 related deaths (35, 36).

Understanding key community members' knowledge, attitudes and beliefs about the novel SARS-CoV-2 virus and whether COVID-19 has been associated with perceived stigma, stigma experiences, or internalized stigma are critical steps for increasing accurate public health knowledge, encouraging uptake of preventative or mitigating health behaviors, including potential uptake of vaccines, and ultimately bringing the COVID-19 pandemic under control. Here, we sought to assess the presence of COVID-19-related stigma in the community, as reported by primary and secondary school teachers in western Kenya in the early phases of the global COVID-19 pandemic, within a cohort of teachers and adolescents already enrolled in a study examining HIV-related stigma and community and individual beliefs related to HIV.

## Materials and Methods

### Study Design

We conducted a one-time, phone-based assessment of perceived, community-based COVID-19 stigma reported by Kenyan primary and secondary school teachers, as well as adolescents living with HIV, utilizing a self-report questionnaire that had been developed and validated for measurement of experienced, perceived, and internalized HIV-related stigma in this setting (37).

The participants were all enrolled in an ongoing, cluster-randomized trial to evaluate whether a multi-media teacher training could decrease Kenyan teachers' negative attitudes and beliefs around HIV and therefore reduce the HIV stigma in their teaching and classrooms (38). The content of the training intervention was focused solely around HIV and increasing empathy toward those living with HIV. Teachers from 20 schools (10 primary schools and 10 secondary schools) in the Turbo Sub-County in western Kenya, were randomized to either an intervention or control group. The intervention group had their HIV-related knowledge, attitudes and beliefs (K/A/B) evaluated immediately before the training, immediately after the training and then at 6 months after the intervention. The control group teachers had their HIV-related K/A/B evaluated at baseline and then at 6 months. Adolescents living with HIV (ALWH) receiving HIV care within the Academic Model Providing Healthcare (AMPATH) program in Kenya and who were also students in any of the 20 schools in the Turbo Sub-County were also enrolled in the study separately for an exploratory evaluation of their experiences and perceptions of HIV-related stigma at 6-months post-teacher intervention. Both the baseline assessments and the training for the intervention group were delivered prior to the emergence of the novel SARS-CoV-2 virus.

This COVID-19 stigma assessment was added onto the parent study's 6-month assessments for teachers in both the intervention and control groups as well as the ALWH. At the time of this COVID-focused stigma assessment, the schools were closed because of the pandemic and the teachers were at home in their communities. The Stigma in AIDS Family Inventory (SAFI) Questionnaire (37) asks a series of questions regarding the stigma and discrimination that an individual experiences because of his/her own or someone in their families' HIV status. The tool has been previously validated for use with Kenyan youth living with HIV and caregivers of children or youth living with HIV (37). Participants are instructed to respond to an experience as having “never happened” or “ever happened.” Many of the question prompts are led by the statement, “Because I have HIV or someone else in my family has HIV…” For this study, we added a new “COVID-version” of the SAFI Stigma Questionnaire, with items modified to ask similar questions regarding stigma and discrimination experiences, but in the context of COVID-19 infection. We analyzed the COVID-19 stigma assessment data by teacher intervention group from the parent study in order to better understand the impact that the multi-media teacher training to mitigate HIV related stigma had on COVID-19 stigma.

### Setting

The research team operates out of the Academic Model Providing Access to Health care (AMPATH) program in Uasin Gishu County, located in western Kenya. Teacher participation in the parent study took place at the primary and secondary schools at which they were employed, or at the AMPATH Chandaria research center in Eldoret, Kenya. The COVID-19 stigma assessment took place over the phone, when both teacher participants and adolescents were at home.

### Data Collection and Analysis

This one-time assessment was completed over the phone between June and November 2020 in order to maintain COVID-19 safety precautions. The study team contacted participants via phone, described the reasons for conducting the additional survey, and asked if the participant was interested and available to complete the assessment. Phone-based consent was obtained from all participants. For those under the age of 18, caregivers provided phone-based consent for adolescents and minors provided assent. The interview was conducted at an appropriate time agreed upon by the participants and study team. Data collected was entered into a REDCap database for analysis. Descriptive analyses were conducted to summarize the perceived and enacted stigma experienced by participants.

### Ethical Approvals

This COVID-19 stigma assessment was added as a modification to the parent study. The additional assessment was approved by the Icahn School of Medicine at Mount Sinai Institutional Review Board, New York, NY, USA, and the Moi University / Moi Teaching and Referral Hospital's Institutional Research and Ethics Committee in Eldoret, Kenya (Approval No: 0003118). Additional approval was received by the National Commission for Science, Technology, and Innovation (NACOSTI), a Kenyan government research regulatory body.

## Results

### Participant Demographics

We recruited and enrolled a total of 330 participants to complete the COVID-19 stigma assessment (99.7% of participants enrolled in the parent study), including 311 primary and secondary teachers (56% female, median age 35 years) and 19 adolescents living with HIV (57.89% female, median age 15 years).

### Responses to Stigma Questionnaire

Responses to stigma items, broken down by cohort, are provided in Table 1. A substantial percentage of teachers (47.8%) reported having witnessed discrimination against someone with suspected COVID-19 infection. A smaller, but sizable number of teacher participants (18%) had experienced COVID-19-related discrimination themselves, and among those who did, most experienced it within their neighborhoods (71.4%). Some teacher participants had lost financial (11.6%) or social (14.8%) support as a result of COVID-19 infection. Over half of the teachers (69.2%) reported that it was important for one to keep their COVID-19 status a secret. The teachers reported a great deal of perceived social stigma related to COVID-19 into the community; the majority of teachers reported that either a few or many people in the community considered COVID-19 “a dirty or shameful disease,” or believed that “students who are suspected to have had COVID-19 infection should not be allowed to return to school.”

**Table 1:** COVID-19 stigma survey responses.

**Statement**	**Teacher cohort *N* (%)**	ALWH^*^ cohort *N* (%)
I have witnessed discrimination against someone with COVID-19 infection	146 (46.8%)	0
I have experienced discrimination	56 (18%)	0
I have experienced discrimination at home	12 (21.4%)	0
I have experienced discrimination in neighborhood	40 (71.4%)	0
I have experienced discrimination in school	0	0
I have experienced discrimination in religious place	0	0
I have experienced discrimination at work	1 (1.78%)	0
I have experienced discrimination at the clinic	2 (3.57%)	0
I have experienced discrimination at another place	2 (3.57%)	0
Lost financial support due to COVID-19	36 (11.6%)	0
Lost social support due to COVID-19	46 (14.8%)	0
Feel it is important to keep COVID-19 status secret	216 (69.2%)	5 (26.32%)
**People in my community think that COVID-19 is a 'dirty' or 'shameful' disease**		
No one thinks that	101 (32.3%)	9 (47.37%)
A few people think that	137 (43.9%)	7 (36.84%)
Most people think that	74 (23.7%)	3 (15.79%)
**Students who are suspected to have had COVID-19 infection should not be allowed to return to school**		
No one thinks that	95 (30.4%)	3 (15.79%)
A few people think that	144 (46.2%)	9 (47.37%)
Most people think that	73 (23.4%)	7 (36.84%)

* *Adolescents living with HIV*.

Among the small sample of ALWH (*N* = 19), none reported witnessing or experiencing COVID-19 related discrimination, and they had not lost financial or social support as a result of COVID-19 infection. Over a quarter (26.32%) of adolescents reported that it was important to keep their COVID-19 status a secret, and just over half of the adolescents reported that a few or most people in the community think “that COVID-19 is a dirty or shameful disease.” Nearly half (47.37%) of adolescents responded “a few people think that” in regard to the statement “students who are suspected to have had COVID19 infection should not be allowed to return to school.”

### COVID-19 Stigma Experiences by Teacher Intervention Group

We stratified responses to the COVID-19 stigma assessment by teacher intervention group to explore whether the prior intervention aiming to reduce HIV stigma in the knowledge, attitudes and beliefs among the teachers also resulted in any reduction in their COVID-19 stigma responses. The intervention group was significantly (*p* = 0.0415) more likely to respond “nobody thinks that” to the statement “People in my community think that COVID-19 is a ‘dirty' or 'shameful disease” as compared to the control group (*N* = 68, 37.2% vs. *N* = 33, 25.8%, respectively). We also saw a statistically significant (*p* = 0.0074) difference in the response to the statement “I feel it is important to keep COVID-19 infection status a secret from other people,” with teachers in the intervention group less likely to respond in agreement than the control group (*N* = 28, 21.9% vs. *N* = 68, 37.2%, respectively).

## Discussion

Our findings, representing one of the first community assessments related to COVID-19 stigma in a low and middle- income country (LMIC), suggest that COVID-19-related stigma may be quite common in western Kenya. The impact of stigma related to infectious and non-communicable diseases is well-studied, but the impact of stigma related to the novel coronavirus remains unknown. A sizable percentage of Kenyan teachers had experienced COVID-19-related discrimination or associated losses of support themselves, and nearly half reported witnessing discrimination against others. Primary and secondary school teachers represent an important stakeholder group within communities as they are both responsible for conveying knowledge to their students and may also significantly shape their attitudes and beliefs, including whether those beliefs are stigmatizing or not. Moreover, teachers tend to be respected and relatively well-educated community members, who also have substantial insight into the psychosocial contexts of the families of the students they teach. In the context of the COVID-19 pandemic, where social distancing and the ability for schools to remain open have been critical questions, teachers may also be particularly attuned to the community and political attitudes shaping these decisions as they have immediate impact on their own work and their perceived risks. The findings here suggest that, while the teachers may not endorse these stigmatizing beliefs themselves, many of them were already witnessing stigmatizing beliefs and behaviors related to COVID-19 in the community.

We observed differences in responses between the teacher participants and adolescents living with HIV. None of the adolescent participants reported witnessing or experiencing discrimination related to COVID-19, nor did they report losing financial and/or social support. This is in contrast to the reports from the teacher participants, demonstrating varying levels of stigma experiences. This evaluation only included a small sample size of adolescents living with HIV, who were students in the classroom of enrolled teacher participants, which may impact these findings. Moreover, the closing of schools and increased isolation due to the pandemic may have impacted adolescents' perception of COVID-19 stigma as they may not have been in the community setting where stigmatizing behaviors or conversations regarding the pandemic are likely to occur. Lastly, as this was a phone-based assessment and most adolescents do not have their own phones in this setting, the adolescents may have been responding to questions in close proximity to caregivers or other community members, potentially impacting their ability to speak openly.

This evaluation was embedded in a cluster-randomized trial of an intervention designed to reduce HIV-related stigma among Kenyan primary and secondary schools. In addition to wanting to describe the emerging beliefs and attitudes related to COVID-19, we also wanted to explore whether any potential impact from an intervention to reduce HIV-related stigma might carry over in regards to this different, novel infectious disease. We did observe significant differences in COVID-19 stigma responses when stratified by teacher intervention group. Teachers in the intervention group, who completed the multi-media training on HIV related stigma were less likely to think that the community views COVID-19 as a dirty or shameful disease, and less likely to feel it was important to keep their COVID-19 infection a secret, than the teacher control group. A key component of the multi-media teacher training was the review and debunking of common myths and misconceptions related to HIV. In this portion of the training, participants were given a pamphlet with common myths and misconceptions, for which the facilitators reviewed and provided clarification to the group. Intervention teachers were given the opportunity to ask questions on common myths in order to allow for discussion of accurate information. This training may have improved the teachers' ability to identify myths, like that COVID-19 is “shameful” or “dirty.” The teacher training also included a session with a person living with HIV sharing their experiences and providing the teachers with a window into the personal impact that HIV-related stigma could have on an individual. An adolescent peer-navigator living with HIV on the study team was present to speak to the teacher-participant group about their own experiences, facilitate conversation as appropriate and answer questions from the group. These shared stories and experiences may have had the desired effect of humanizing the HIV experience among the intervention group, which could have transferrable effects related to the COVID-19 experience, thus influencing one's response to “I feel it is important to keep COVID-19 infection status a secret from other people.” These data offer the possibility that the skills attained during the HIV stigma training, whether in sorting out misinformation or responding empathetically to persons living with an infectious disease, could be transferrable to other infectious disease outbreaks.

## Limitations

There are a number of important limitations for this study to consider. First, this study included a small sample of primary and secondary school teachers and adolescents living which HIV, and was not designed to be representative of the general community. Second, teacher participant enrollment in the parent HIV-stigma training may have impacted the overall results of this COVID-19 stigma assessment, and therefore the prevalence of COVID-19-related stigma with a non-intervention community group could demonstrate much higher rates of stigma. Third, we did not use a validated COVID-19 stigma questionnaire, rather we adapted a rigorously developed and validated HIV stigma scale for this setting. Lastly, the rapidly evolving nature of the COVID-19 pandemic makes understanding related stigma a rapidly moving target, as the introduction of vaccines and other public health measures continue to influence community knowledge, attitudes and beliefs.

## Recommendations and Conclusion

It is critical that these findings are used to shape education strategies that facilitate health behavior changes to mitigate pandemic impact, as they suggest that interventions to reduce community level stigma for HIV may have a protective impact on other stigmatized infectious diseases such as COVID-19. The COVID-19 pandemic highlights the critical importance of engaging marginalized communities in accurate health information. Access to, understanding, and acceptance of accurate information are necessary for a population to rapidly adapt to changing environments or new policies to mitigate the risk of disease (39). Currently, the world faces not just a new global pandemic, but also an “infodemic” with an overabundance of information and dissemination of incorrect information that undermines the public health response (40). Effective communication strategies are necessary to mitigate stigmatizing beliefs and behavior and improve access to care and engagement in COVID-19 preventative behaviors.

## Data Availability Statement

The raw data supporting the conclusions of this article will be made available by the authors, without undue reservation.

## Ethics Statement

The studies involving human participants were reviewed and approved by Icahn School of Medicine at Mount Sinai Institutional Review Board, New York, NY, USA; Moi University/Moi Teaching and Referral Hospital's Institutional Research and Ethics Committee in Eldoret, Kenya; National Commission for Science, Technology, and Innovation (NACOSTI). Written informed consent to participate in this study was provided by the participants' legal guardian/next of kin.

## Author Contributions

RV, WN, and EA designed and provided scientific oversight for the study. Material preparation, data collection and analysis were performed by AC, CA, JA, WB, and DM. The first draft of the manuscript was written by AC and all authors reviewed and contributed to subsequent versions of the manuscript. All authors read and approved the final manuscript.

## Funding

This study was funded by an R21 grant (1R21TW011071-01) to RV from the National Institutes for Health Fogarty International Center. The funders did not participate in the development of the concept, analysis of data or writing of the manuscript. Open access publication fees will be covered by the Arnhold Institute for Global Health at the Icahn School of Medicine at Mount Sinai.

## Conflict of Interest

The authors declare that the research was conducted in the absence of any commercial or financial relationships that could be construed as a potential conflict of interest.

## Publisher's Note

All claims expressed in this article are solely those of the authors and do not necessarily represent those of their affiliated organizations, or those of the publisher, the editors and the reviewers. Any product that may be evaluated in this article, or claim that may be made by its manufacturer, is not guaranteed or endorsed by the publisher.
